# High-resolution entry and exit surface dosimetry in a 1.5 T MR-linac

**DOI:** 10.1007/s13246-023-01251-6

**Published:** 2023-03-29

**Authors:** E. Patterson, P. Stokes, D. Cutajar, A. Rosenfeld, J. Baines, P. Metcalfe, M. Powers

**Affiliations:** 1 Centre of Medical and Radiation Physics, University of Wollongong, Wollongong, NSW Australia; 2 Illawarra Health Medical Research Institute, University of Wollongong, Wollongong, NSW Australia; 3 Ingham Institute for Applied Medical Research, Liverpool, NSW Australia; 4 Townsville Cancer Centre, Townsville Hospital and Health Service, Townsville, QLD Australia; 5 College of Science and Engineering, James Cook University, Townsville, QLD Australia

**Keywords:** MR-linac, Elekta Unity, MOSFET, Skin dose, Surface dosimetry, Monte Carlo

## Abstract

The magnetic field of a transverse MR-linac alters electron trajectories as the photon beam transits through materials, causing lower doses at flat entry surfaces and increased doses at flat beam-exiting surfaces. This study investigated the response of a MOSFET detector, known as the MO*Skin*™, for high-resolution surface and near-surface percentage depth dose measurements on an Elekta Unity. Simulations with Geant4 and the Monaco treatment planning system (TPS), and EBT-3 film measurements, were also performed for comparison. Measured MO*Skin*™ entry surface doses, relative to D_max_, were (9.9 ± 0.2)%, (10.1 ± 0.3)%, (11.3 ± 0.6)%, (12.9 ± 1.0)%, and (13.4 ± 1.0)% for 1 × 1 cm^2^, 3 × 3 cm^2^, 5 × 5 cm^2^, 10 × 10 cm^2^, and 22 × 22 cm^2^ fields, respectively. For the investigated fields, the maximum percent differences of Geant4, TPS, and film doses extrapolated and interpolated to a depth suitable for skin dose assessment at the beam entry, relative to MO*Skin*™ measurements at an equivalent depth were 1.0%, 2.8%, and 14.3%, respectively, and at a WED of 199.67 mm at the beam exit, 3.2%, 3.7% and 5.7%, respectively. The largest measured increase in exit dose, due to the electron return effect, was 15.4% for the 10 × 10 cm^2^ field size using the MO*Skin*™ and 17.9% for the 22 × 22 cm^2^ field size, using Geant4 calculations. The results presented in the study validate the suitability of the MO*Skin*™ detector for transverse MR-linac surface dosimetry.

## Introduction

Megavoltage (MV) photon beams are typically used to treat deep-seated tumours because of the skin-sparing effect. In recent years image-guided radiation therapy (IGRT) on MV photon treatment systems has developed, and online magnetic resonance-guided radiation therapy (MRgRT), offering superior soft tissue contrast imaging, is now available. The delivery of these treatments is facilitated by machines that combine a magnetic resonance (MR) scanner with a linear accelerator (linac), referred to as an MR-linac. These systems offer a non-ionizing form of imaging. One of the two commercial MR-linac systems currently available is the Elekta Unity (Elekta, Stockholm, Sweden) which integrates a 1.5 T magnetic resonance imaging unit and a linac with a flattening-filter-free (FFF) 7 MV photon beam. For these MR-linacs the radiation beam is perpendicular to the magnetic field direction.

The impact of the transverse magnetic field on secondary electron transport is well established in the literature [1–3]. Dose perturbations include a lateral shift in the dose distribution and asymmetric penumbra [1, 4, 5], a reduction in the depth of maximum dose (d_max_) [1, 2, 4–7], and the electron return effect (ERE) [2, 4, 8]. There is also evidence that skin-sparing can be enhanced, compared to conventional linac (0 T) treatments, due to the magnetic field sweeping contaminant secondary electrons away from the treatment area [5–13]. However, in some situations, such as when the entry and exit surfaces are oblique, this is not the case [8, 9, 11], and the corresponding dose due to the ERE is non-trivial [9, 11].

Previous investigations of near-surface dose in a transverse MR-linac have used dosimeters with varying effective point of measurement (EPOM). These include radiochromic film [6, 14, 15], PTW 31,021 Semiflex 3D [16], optically stimulated luminescence dosimeters (OSLDs) [17–19], thermoluminescent dosimeters (TLDs) [3], metal–oxide–semiconductor field-effect transistor (MOSFET) [20], gel [21], PTW 60019 microDiamond [6, 14, 16], and a PTW 34045 Advanced Markus chamber [6, 12]. Parallel-plate chambers, such as the Advanced Markus chamber, are commonly used for dose measurements in the build-up region on conventional linacs. For MR-linac dosimetry, the magnetic field influences charge collection in the air-filled sensitive volume (SV) of the ionization chamber [22]. For reference dosimetry, near constant correction factors, specific to the magnetic field, can be applied to ionisation chamber measurements beyond d_max_; however, in the build-up region correction factors become depth-dependent [23] due to a loss of charged-particle equilibrium (CPE) conditions. With a variable magnetic field correction factor in the build-up region and a SV thickness in the order of millimetres [24], the ionisation chamber is not an ideal dosimeter to accurately measure skin dose in a transverse MR-linac.

According to the International Commission on Radiological Protection (ICRP), 0.07 mm is the recommended skin depth for practical skin dose assessment [25]. This depth corresponds to the well-established average nominal basal layer that is responsible for the production of skin cells and is considered the most radiosensitive (skin) tissue in terms of deterministic and stochastic effects, such as basal cell carcinoma [25]. To accurately measure skin dose, a dosimeter with a small SV and reproducible water equivalent depth (WED) of 0.07 mm should be considered, to avoid volume averaging that can occur in heterogeneous dose regions. Similarly for small-field dosimetry, the size of the SV should be considerably smaller than the field size as a loss of CPE impacts the detectors readings [26]. With skin dose specified at 0.07 mm, previous near-surface dose investigations on a transverse MR-linac are lacking as dosimeters with larger EPOMs were used [6, 14–21, 27]. The dose averaged across the SV of an OSLD corresponds to a near-skin water equivalent depth (WED) of 0.16 mm, with the external casing of the OSLD removed [17]. Similarly, film positioned at the surface has a WED of 0.14 mm (i.e. half the thickness of a sheet of EBT-3 film).

Of interest is a study that investigated surface and near-surface dose measurements, at the beam entry and exit, in a 1.5 T transverse MR-linac using nanoDot OSLDs (Landauer, Glenwood, USA) [17]. The study reported surface doses, relative to the maximum dose (D_max_), of 15.7%, 16.7%, and 18.0%, at the beam entry, and 56.0%, 62.8%, and 63.4% at the beam exit, for 5 × 5 cm^2^, 10 × 10 cm^2^, and 22 × 22 cm^2^ fields, respectively [17]. The authors commented that further experimental investigations were required since previous film data reported entry surface doses of 34.6% and 35.8%, relative to D_max_, for field sizes 5 × 5 cm^2^ and 10 × 10 cm^2^, respectively [16].

For accurate Monte Carlo simulations of skin dose, high-resolution scoring is recommended [8]. Unlike experimental dosimeters with a fixed SV, Monte Carlo simulations can be scored according to the user’s specifications. Dose calculations at the beam entry and exit for a 6 MV linac with a 1.5 T transverse magnetic field, have previously been investigated [8]. Using 0.01 mm thick voxels, Monte Carlo calculations determined a sharp increase in dose from 10.0 to 40.0%, normalised to D_max_, in the first millimetre of the build-up region for a 10 × 10 cm^2^ field. At the beam exit for the same field size, the dose increased from 40.0% to 55.0%, relative to D_max_, as a direct consequence of the ERE [8]. Using high-resolution scoring geometry at the beam entry and exit reveals the extent of the dose gradient that otherwise would be masked using a larger dose voxel size. Likewise, using a dosimeter with a large SV, relative to the steep gradient, would cause volume averaging and inaccurate dosimetry.

We propose using a MOSFET detector, called the MO*Skin*™, for surface dosimetry in a 1.5 T transverse MR-linac. The MO*Skin*™ features a small SV, 4.8 × 10^–6^ mm^3^ [28], which provides high spatial resolution suitable for dosimetry in small fields and high dose gradient regions, including the build-up region of MV x-ray beams. Prior studies have established that the MO*Skin*™ detector is well-suited for implementation across a range of radiation sources including Ir-192 [29], Co-60 [30], electron [31], flattening filter [32–36], and FFF [36–39] beams. MOSFET detectors often have an epoxy bubble to protect the SV; however, the MO*Skin*™ utilises a thin and reproducible polyimide build-up with a water-equivalent thickness of 0.07 mm [32]. These features along with a thin overall thickness of 0.4 mm [28], make the MO*Skin*™ an ideal candidate for skin [25] and surface dosimetry. Another benefit of MOSFET technology is the real-time readout of measurements [32], while dosimeters such as film and OSLD require more stringent preparation and read-out protocols. Additionally, the MO*Skin*™ reportedly experiences no significant readout changes in the presence of a 1.0 T static magnetic field [40]. Previous MR-linac measurements with the MO*Skin*™ on a 1.0 T inline MR-linac show comparable results to Geant4 simulations, film, and a microDiamond detector, demonstrating the suitability of the detector in an MR environment [38, 39, 41]. Readers wishing for greater detail on the mechanisms behind general MOSFET and MO*Skin*™ dosimetry readout are referred elsewhere [42].

The objective of this study is to use the MO*Skin*™ to experimentally characterise surface and near-surface dose on a 1.5 T transverse MR-linac. The term ‘skin dose’ will herein be synonymous with ‘surface dose’ [43], measured on a water phantom, and measurement points beyond a surface depth of 0.07 mm will be referred to as ‘near-surface dose’. Based on our review of available literature, this would be the first published work of MOSFET’s in a 1.5 T transverse MR-linac and the first published work of experimental skin dose, at a depth of 0.07 mm, in a 1.5 T transverse MR-linac. Experimental measurements with Gafchromic EBT-3 film (Ashland ISP Advanced Materials, NJ, USA) and simulations were also performed to compare to the MO*Skin*™ measurements.

## Methodology

### Measurements

#### MO*Skin*™ calibration

MO*Skin*™ detectors were calibrated on the Elekta Unity using the 7 MV FFF photon beam with a source-isocentre-distance (SID) of 143.5 cm, which coincides with a position 14.0 cm above the surface of the treatment couch. A 1.0 cm-thick custom-milled solid water (Gammex Solid Water-Model # 457, Middleton, WI) holder was manufactured such that the MO*Skin*™ was flush at the surface and without air gaps when placed at depth. The detectors were each placed face-up at the machine isocentre, at a 5.0 cm depth within a 15.0 × 30.0 × 30.0 cm^3^ solid water phantom, at a source-surface-distance (SSD) of 138.5 cm. For face-up MO*Skin*™ orientation, the WED of the SV is 0.07 mm whereas, with a face-down orientation, the WED of the SV is approximately 0.9 mm [32].

**Fig. 1:**
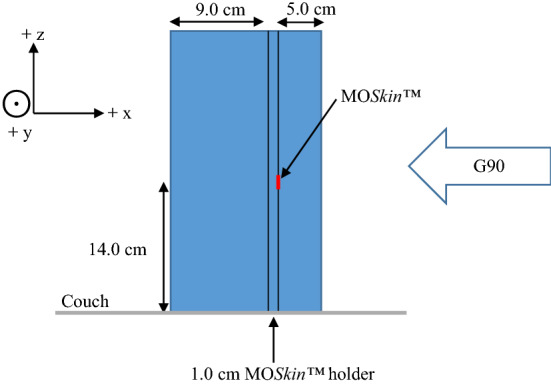
Schematic of the set-up used to calibrate the MO*Skin*™ detectors. The MO*Skin*™ was placed at the machine isocentre 14.0 cm above the couch surface, and at a depth of 5.0 cm. The IEC-61217 coordinate system is shown in the top left of the image. The arrow with ‘G90’ inside, indicates the beam direction and B_0_ is directed out of the page, parallel to the y-axis

The experimental set-up was verified using the megavoltage imager (MVI) panel that is integrated into the Elekta Unity system, and a machined aluminium ruler. This ruler consists of thirteen machined 3.0 mm circular holes along the central axis at 2.0 cm intervals. The ruler was placed on top of the phantom blocks aligned with the y-axis. A schematic of the MO*Skin*™ calibration set-up with the coordinate system is shown in Fig. 1. Care was taken to reduce air gaps between the phantom slabs to minimise ERE. Using a 10 × 10 cm^2^ field, gantry 90.0° (G90), 100 MU was delivered three times for each MO*Skin*™. G90 was used to avoid issues related to the variation of the helium level inside the cryostat. The average MO*Skin*™ read-out was calculated and used to determine a unique calibration factor for each detector to relate the threshold voltage shift to the dose deposited within the SV [32]. This Elekta Unity system is calibrated to give 1.0 Gy/100 MU to the isocentre at a depth of 5.0 cm in water, 138.5 cm SSD, for a 10 × 10 cm^2^, G90, field.

#### Film calibration

EBT-3 film calibration was performed using 2.0 × 4.0 cm^2^ film strips within a 30.0 × 30.0 × 19.0 cm^3^ solid water phantom. Films were positioned at 5.0 cm depth within the phantom and irradiated with a G0 10 × 10 cm^2^ field for 0, 100, 200, 400, and 800 MU. Films were scanned and digitised using an Epson Expression 12000XL flatbed scanner (Seiko. Epson Corporation, Nagano, Japan) in transmission mode, without colour correction and with a scan resolution of 75 DPI (0.34 mm pixel size), using 48-bit RGB mode. Multichannel analysis was used within the FilmQA™ Pro software (Ashland ISP Advanced Materials, NJ, USA) and lateral scanner variations were corrected using MATLAB (MathWorks Inc., MA) [44]. A central 1.0 × 1.0 cm^2^ region of interest (ROI) on each film was used to correlate mean pixel values with the delivered dose.

#### MO*Skin*™ PDD

**Fig. 2:**
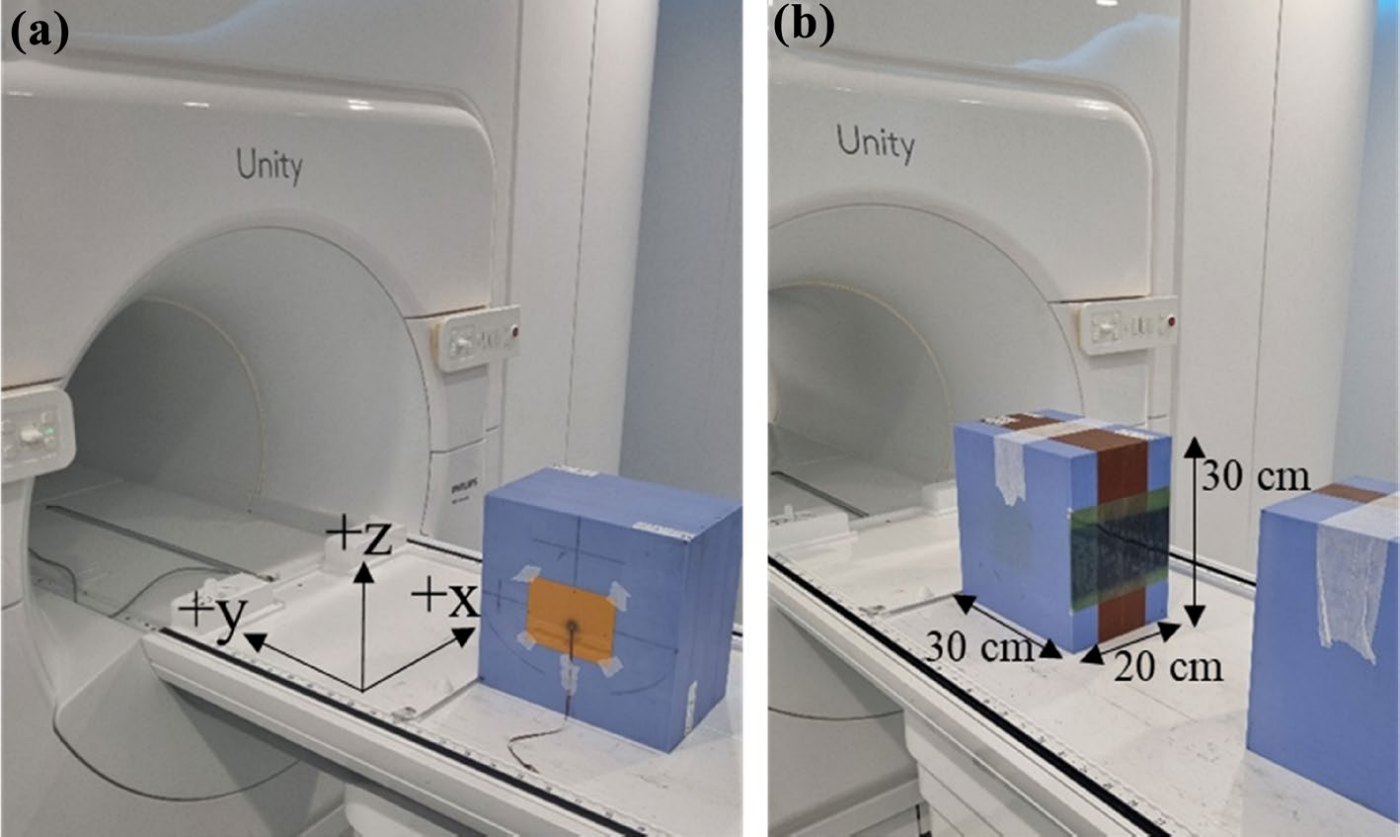
**a** MO*Skin*™ positioning verified using RTQA2 film at the exit surface of the 20.0 × 30.0 × 30.0 cm^3^ solid water phantom for a 1 × 1 cm^2^ field size. **b** EBT-3 film placement for depth dose measurements. Water was applied to both sides of the film to minimise air bubbles between the solid water blocks

In-field percentage depth dose (PDD) curves were measured in a 20.0 × 30.0 × 30.0 cm^3^ solid water phantom, containing the MO*Skin*™ and holder, with the phantom centre coincident with the isocentre. A face-up MO*Skin*™ orientation, relative to the primary beam direction, was used for all measurements. Due to the thin SV of the MO*Skin*™ device, volume averaging can be considered negligible and at the surface, the EPOM is equivalent to a WED of 0.07 mm. For consistency, all MO*Skin*™ measurement depths were expressed in terms of WED. Phantom dimensions were chosen to match the set-up from a previous investigation [17]. Again, the MVI and aluminium ruler were used for the phantom set-up. RTQA2 film (Ashland ISP Advanced Materials, NJ, USA) was used to verify the final alignment before measurements (Fig. 2a). For this study, entry and exit surfaces were flat and perpendicular to the beam direction to avoid significant dose variations that occur at oblique surfaces due to the ERE [11].

Depth dose measurements were acquired for 1 × 1 cm^2^, 3 × 3 cm^2^, 5 × 5 cm^2^, 10 × 10 cm^2^, and 22 × 22 cm^2^ fields at G90 and 133.5 cm SSD. Readings in the build-up region were acquired at 0.07 mm, 0.12 mm, 0.17 mm, and 0.27 mm WEDs, with varying amounts of Polyimide (Kapton) tape [0.05 mm water equivalent thickness (WET)] over the detector surface for each measurement. Note that for photons energies > 1 MeV, the mass attenuation coefficient of Kapton and water closely match [45, 46]. In addition to the above, the MO*Skin*™ was also placed at physical depths of 1.0 mm, 3.0 mm, 5.0 mm, 10.0 mm, and 13.0 mm that correspond to WEDs, relative to the primary beam direction, of 1.07 mm, 3.07 mm, 5.07 mm, 10.07 mm, and 13.07 mm, respectively. For exit beam measurements, the MO*Skin*™ was placed at physical depths of 186.6 mm, 189.6 mm, 194.6 mm, 196.6 mm, 198.6 mm, and 199.6 mm, corresponding to WEDs of 186.67 mm, 189.67 mm, 194.67 mm, 196.67 mm, 198.67 mm, and 199.67 mm, respectively. For each depth, three measurements were acquired to obtain an average dose and standard deviation value. All dose measurements were normalised relative to the reading at d_max_, approximately 13.0 mm for the Elekta Unity system [16], for each respective field. The uncertainty of normalised MO*Skin*™ doses was calculated using the relative uncertainty of the respective depth dose and d_max_ dose measurement for the same field size, that were added in quadrature. The relative uncertainty for each measurement was estimated based on the standard deviation of the mean of three repeated measurements.

#### Film PDD

PDD measurements with EBT-3 film required the use of an extended solid water phantom where film, 20.32 cm in length, was taped along the 20.0 cm phantom surface edge and sandwiched between an opposing solid water stack (Fig. 2b). This orientation had the long axis of the film parallel to the beam direction, G90, to record depth dose values with the fewest number of measurements. Both sides of the film were sprayed with water to minimise air gaps and reduce dose perturbations arising from ERE. The same fields as above were delivered with 500 MU. To avoid effects from film overhang at either surface, two films were individually irradiated for each field such that for one the film edge was flush with the phantom entry and similarly at the exit for the second film. Cutting the 20.32 cm film to a length of 20.0 cm was not an option as this delaminates the film and would render the dose near the cut edge inaccurate. After irradiation, films were scanned and analysed using the same method as above for calibration. Profiles along the central axis, averaged across 10 pixels, for each field size were acquired and normalised to the reading at d_max_. Due to scan resolution, film entry and exit measurement points begin at a depth of 0.34 mm within the solid water phantom. Film dosimetry used in this investigation followed a protocol that yielded a dose uncertainty below 3.0% [47].

### Simulations

#### Geant4

Simulations of the experimental geometry were performed using Geant4 and the Monaco treatment planning system (TPS) for comparison with measured data. The EPOM of Geant4 and Monaco TPS data corresponds to the centre of each scoring voxel. When comparing the simulation to experimental results, the WED to the centre of the respective voxel was used. Using Geant4 version 11.0.2, a 20.0 × 30.0 × 30.0 cm^3^ water equivalent block phantom (*ρ* = 1.00 g/cm^3^) was created. With an SSD of 133.5 cm, beams were fired from phase space files (provided by Elekta) with a total of 1.12 × 10^10^ primary histories per field size and a dose uncertainty of approximately ± 0.4%. As specified in supporting documentation provided by Elekta, each phase space plane was located 129.5 cm from the source. The maximum step length was set to 0.01 mm. The dose within the phantom was scored using a 0.1 × 1.0 × 1.0 mm^3^ dose grid, where the dose along the direction of the beam (x-axis) was sampled at 0.1 mm increments. When determining depth dose profiles, adjacent voxels surrounding the central axis of the beam were averaged. For the smallest field size, 1 × 1 cm^2^, two dose voxels in the y and z axes were averaged. This was scaled for each field size such that for the 22 × 22 cm^2^, 44 dose voxels in the y and z axes were averaged. For each beam, the depth dose curve was normalised to the reading at d_max._

Monaco TPS.

For Monaco computations, a CT image of air was acquired with a 1.0 mm slice thickness and imported into the Monaco TPS (v5.4). A 20.0 × 30.0 × 30.0 cm^3^ solid volume was contoured, centred to the isocentre, and set as the external structure. A forced relative electron density (RED) of 1.000 was applied to the contoured volume and couch structures were added. 1 × 1 cm^2^, 3 × 3 cm^2^, 5 × 5 cm^2^, 10 × 10 cm^2^, and 22 × 22 cm^2^ 500 MU, G90, beams were added. Calculations were performed using a 0.1 cm dose grid and a statistical uncertainty of 0.2% per control point. A transverse dose plane, centred at the isocentre, was exported for each field size. Dose planes were imported into Verisoft v7.2(PTW-Freiburg, Germany) where profiles were extracted. For each beam, the depth dose curve was normalised to the reading at d_max._

Cubic piecewise polynomials were used to extrapolate and interpolate measured and simulated doses to equivalent WEDs using MATLAB’s inbuilt function, ‘*spline*’. The fitting of cubic splines for the film, Geant4, and Monaco datasets were used to generate PDD graphs. Comparisons between normalised datasets were made using percent difference (Eq. 1) and percent change (Eq. 2).

1 $$\% Difference=a-b$$

2 $$\% Change=\frac{a-b}{a}\times 100\%$$

## Results

### Experimental surface and near-surface dose measurements with MO*Skin*™ and film

**Fig. 3:**
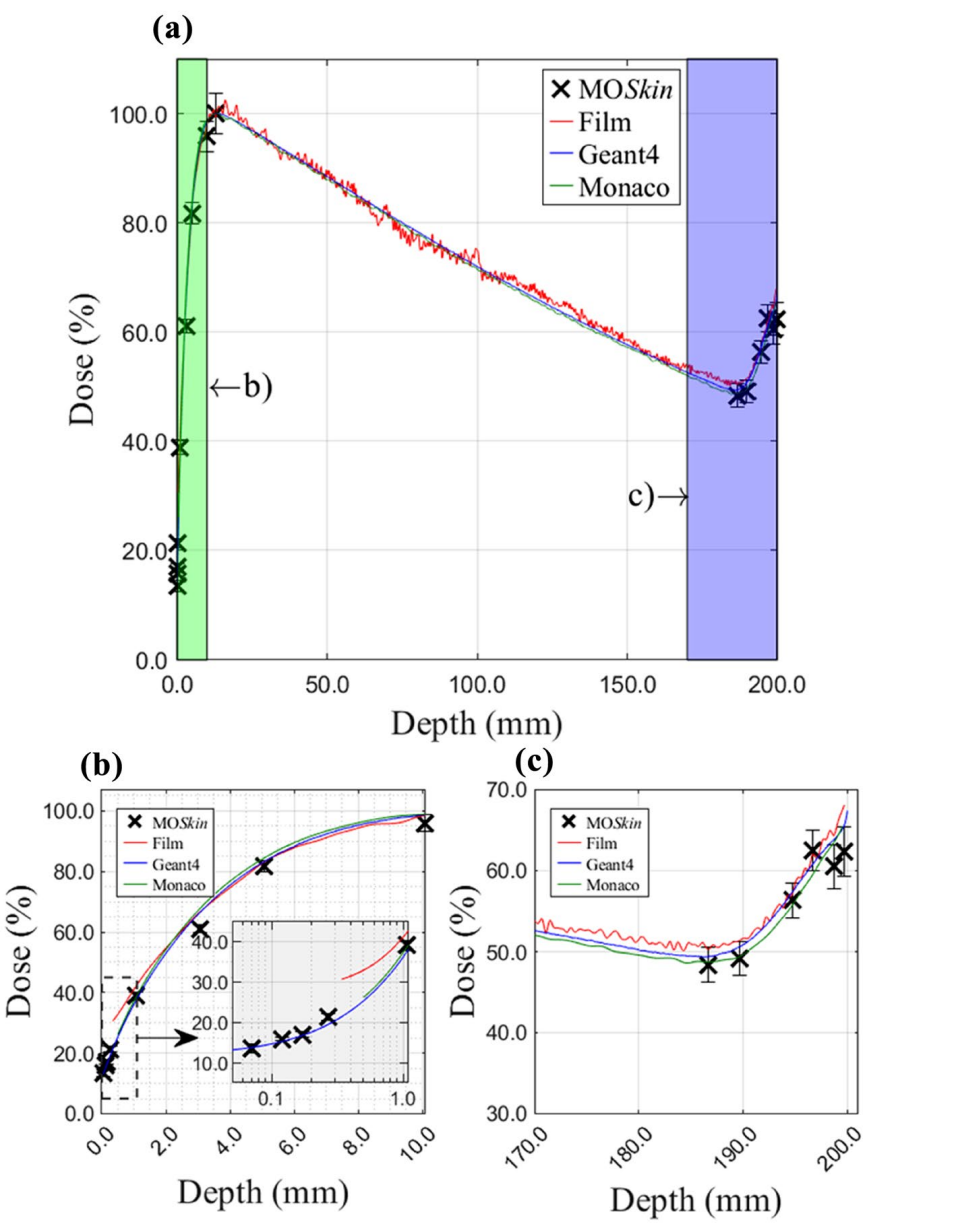
**a** Full length experimental and simulation phantom PDDs, normalised to D_max_, for the 22 × 22 cm^2^ field size. The green and purple shaded regions in **a **are magnified in **b** and **c**, for clarity and represent the first 10.0 mm and last 30.0 mm depth of the PDD. An inset plot in **b** uses a log scale for the x-axis to resolve the data points contained within the first millimetre of the build-up region

**Fig. 4:**
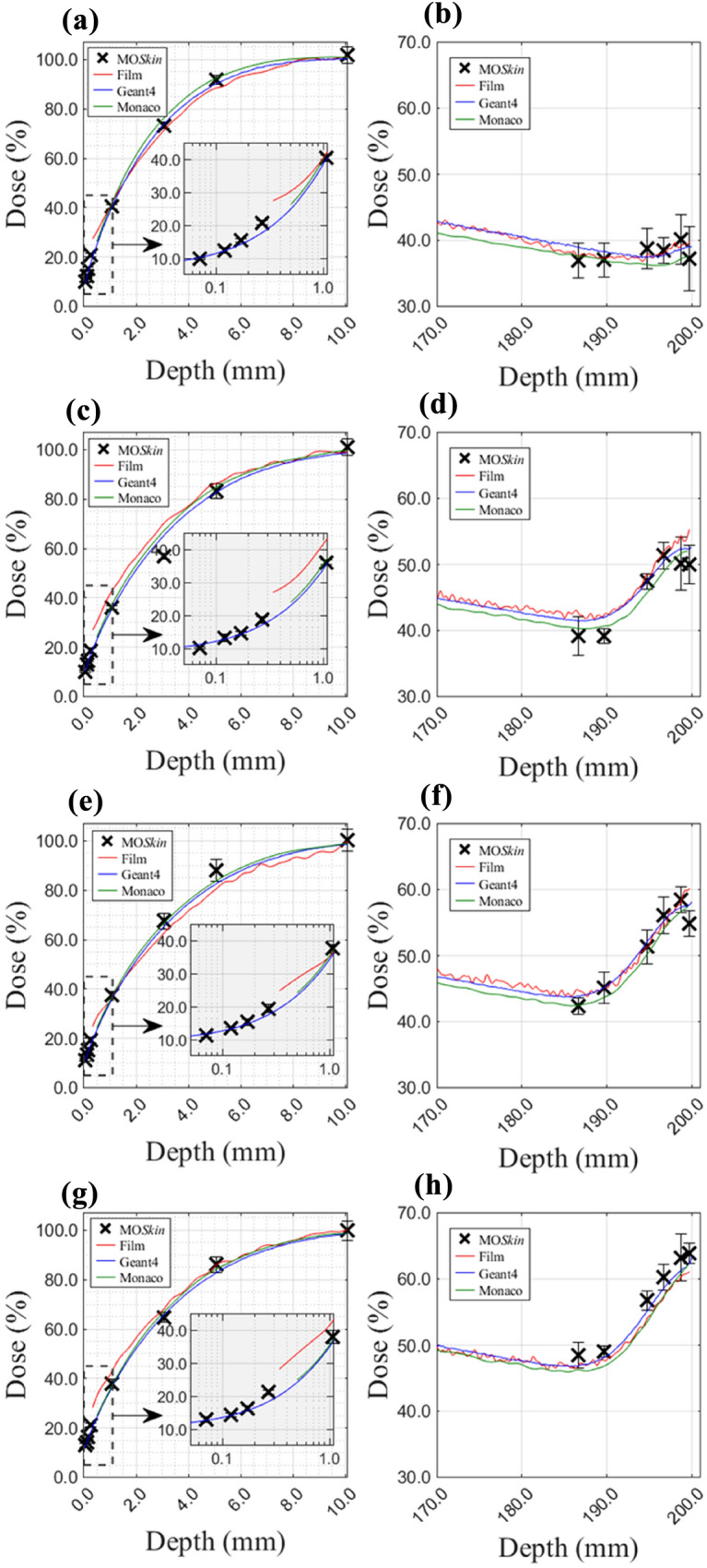
Magnified PDDs, normalised to D_max_, with Monaco TPS and Geant4 calculations overlaid with experimental MO*Skin*™ and film measurements. Subplots **a**, **c**, **e**, and **g** show entry PDDs for the first 10.0 mm with the addition of an inset plot (x-axis log scale) for field sizes 1 × 1, 3 × 3, 5 × 5, and 10 × 10 cm^2^. Subplots **b**, **d**, **f**, and **h** show exit PDDs in the last 30.0 mm of the phantom for the same fields

Calibration factors determined for the two MO*Skin*™ detectors used in this work were (2.22 ± 0.03) mV/cGy and (2.22 ± 0.01) mV/cGy. The 22 × 22 cm^2^ field size PDD is presented in Fig. 3a with entry and exit regions highlighted in Fig. 3b and c, respectively. Entry and exit PDDs for field sizes 1 × 1 cm^2^, 3 × 3 cm^2^, 5 × 5 cm^2^, and 10 × 10 cm^2^ are plotted in Fig. 4.

Calculated, measured, and spline-estimated build-up region doses are summarised in Table 1. MO*Skin*™ surface doses, 0.07 mm WED, at the beam entry were (9.9 ± 0.2)%, (10.1 ± 0.3)%, (11.3 ± 0.6)%, (12.9 ± 1.0)%, and (13.4 ± 1.0)% for 1 × 1 cm^2^, 3 × 3 cm^2^, 5 × 5 cm^2^, 10 × 10 cm^2^, and 22 × 22 cm^2^ field sizes, respectively. Measured near-surface film doses, at a depth of 0.34 mm along the PDD, were 27.4%, 26.9%, 24.7%, 28.1%, and 30.5% for the same field sizes with percent differences ranging between 13.5% and 17.5% to 0.07 mm WED MO*Skin*™ surface doses. At a 0.27 mm WED, the MO*Skin*™ measured doses were (20.8 ± 0.7)%, (18.7 ± 1.0)%, (19.3 ± 1.2)%, (21.1 ± 0.8)%, and (21.3 ± 0.9)% for 1 × 1 cm^2^, 3 × 3 cm^2^, 5 × 5 cm^2^, 10 × 10 cm^2^, and 22 × 22 cm^2^ field sizes, respectively, and percent differences to measured 0.34 mm WED film doses varied between − 5.4% and − 9.2%. MO*Skin*™ doses interpolated to 0.34 mm at the beam entry showed improved agreement to measured film doses at an equivalent depth with percent differences between − 2.8% and − 6.0% across all field sizes. Film doses extrapolated to a 0.07 mm WED showed variable agreement, between 2.0 and 14.3% (percent difference), to skin-dose equivalent MO*Skin*™ measurements.

**Table 1:** Comparison of surface and near-surface doses of the MO*Skin*™ detector with Geant4 and Monaco TPS calculations at the beam entry of the phantom

Field size (cm^2^)	Entry (% D_max_)				
	Depth	MO*Skin*™	Film	Geant4	Monaco TPS
1 × 1	0.05 0.07 0.34 0.50 1.07	9.2 (X) 9.9 ± 0.2 24.0 (I) 29.7 (I) 40.3 ± 0.9	23.0 (X) 23.2 (X) 27.4 30.3 (I) 41.8 (I)	9.3 ± 0.4 10.2 (I) 20.1 (I) 25.0 (I) 40.1 (I)	12.0 (X) 12.7 (X) 21.4 (X) 26.3 ± 0.2 41.7 (I)
3 × 3	0.05 0.07 0.34 0.50 1.07	8.0 (X) 10.1 ± 0.3 21.6 (I) 27.0 (I) 36.1 ± 0.8	23.7 (X) 23.8 (I) 26.9 30.2 (I) 42.8 (I)	10.5 ± 0.4 11.2 (I) 19.0 (I) 23.0 (I) 35.3 (I)	11.8 (X) 12.4 (X) 19.9 (X) 24.0 ± 0.2 37.0 (I)
5 × 5	0.05 0.07 0.34 0.50 1.07	10.4 (X) 11.3 ± 0.6 21.9 (I) 26.8 (I) 37.5 ± 1.3	12.2 (X) 13.4 (X) 24.7 28.6 (I) 36.2 (I)	11.1 ± 0.4 11.7 (I) 19.5 (I) 23.4 (I) 35.7 (I)	12.6 (X) 13.2 (X) 20.3 (X) 24.2 ± 0.2 36.8 (I)
10 × 10	0.05 0.07 0.34 0.50 1.07	12.5 (X) 12.9 ± 1.0 24.3 (I) 29.9 (I) 37.9 ± 2.0	13.6 (X) 14.9 (X) 28.1 32.9 (I) 42.7 (I)	12.0 ± 0.4 12.6 (I) 20.2 (I) 24.2 (I) 36.3 (I)	14.4 (X) 14.9 (X) 21.3 (X) 24.9 ± 0.2 36.9 (I)
22 × 22	0.05 0.07 0.34 0.50 1.07	11.6 (X) 13.4 ± 1.0 24.6 (I) 30.3 (I) 38.9 ± 1.3	27.6 (X) 27.7 (X) 30.5 32.8 (I) 42.1 (I)	13.1 ± 0.4 13.7 (I) 21.3 (I) 25.2 (I) 37.4 (I)	14.3 (X) 14.9 (X) 22.1 (X) 26.1 ± 0.2 38.5 (I)

Quoted doses are normalised to the reading at d_max._ Cubic spline polynomials were used to generate extrapolated and interpolated doses, which are indicated by the symbols (X) and (I), respectively

Similarly, doses obtained from measurements, calculations, and spline estimations at the exit surface are presented in Table 2. MO*Skin*™ doses at the beam exit surface with a 199.67 mm WED, were (37.2 ± 4.9)%, (50.0 ± 2.9)%, (54.9 ± 2.0)%, (63.9 ± 1.6)% and (62.4 ± 3.0)% for the 1 × 1 cm^2^, 3 × 3 cm^2^, 5 × 5 cm^2^, 10 × 10 cm^2^, and 22 × 22 cm^2^ field sizes, respectively. MO*Skin*™ doses interpolated to a 199.66 mm WED, suitable for direct depth dose comparisons to measured film results, were found to be consistent with measured 199.67 mm WED MO*Skin*™ data for all fields except the 5 × 5 cm^2^. The MO*Skin*™ dose, when measured at a WED of 199.67 mm for the 5 × 5 cm^2^ field size, was found to be comparatively lower than both the exit dose obtained from film measurements and the estimated exit surface doses obtained through Geant4 and Monaco TPS calculations. As a result of this discrepancy, the MO*Skin*™ data point was deemed an outlier and excluded from the sample points used for extrapolation and interpolation fits. Hence, the 199.66 mm WED MO*Skin*™ interpolated dose differs from the 199.67 mm WED MO*Skin*™ measurement for the 5 × 5 cm^2^ field size. At a WED of 199.66 mm, exit beam surface doses measured with film were 39.8%, 55.3%, 60.2%, 61.1%, and 68.1% for the 1 × 1 cm^2^, 3 × 3 cm^2^, 5 × 5 cm^2^, 10 × 10 cm^2^, and 22 × 22 cm^2^ field sizes, respectively. Additionally, the maximum percent difference between measured exit film doses and interpolated MO*Skin*™ data at an equivalent WED was 5.8%, for all fields investigated.

**Table 2:** Comparison of surface and near-surface doses of the MO*Skin*™ detector with Geant4 and Monaco TPS calculations at the beam entry of the phantom.

Field size (cm^2^)	Entry (% D_max_)				
	Depth	MO*Skin*™	Film	Geant4	Monaco TPS
1 × 1	199.50 199.66 199.67 199.95	37.7 (I) 37.2 (I) 37.2 ± 4.9 36.4 (X)	39.5 (I) 39.8 39.8 (X) 41.1 (X)	39.0 (I) 39.0 (I) 39.0 (I) 39.1 ± 0.4	37.5 ± 0.2 37.5 (X) 37.2 (X) 37.5 (X)
3 × 3	199.50 199.66 199.67 199.95	49.9 (I) 50.0 (I) 50.0 ± 2.9 50.3 (X)	54.5 (I) 55.3 55.4 (X) 56.6 (X)	52.3 (I) 52.4 (I) 52.4 (I) 52.6 ± 0.4	52.0 ± 0.2 52.0 (X) 52.0 (X) 51.8 (X)
5 × 5	199.50 199.66 199.67 199.95	59.4 (I) 59.6 (I) 54.9 ± 2.0 60.0 (X)	59.9 (I) 60.2 60.2 (X) 62.2 (X)	57.6 (I) 57.7 (I) 57.7 (I) 58.2 ± 0.4	57.4 ± 0.2 57.6 (X) 57.6 (X) 57.9 (X)
10 × 10	199.50 199.66 199.67 199.95	63.8 (I) 63.9 (I) 63.9 ± 1.6 63.9 (X)	60.9 (I) 61.1 61.1 (X) 61.8 (X)	62.1 (I) 62.4 (I) 62.4 (I) 63.4 ± 0.4	62.1 ± 0.2 62.3 (X) 62.3 (X) 62.8 (X)
22 × 22	199.50 199.66 199.67 199.95	61.7 (I) 62.4 (I) 62.4 ± 3.0 64.0 (X)	67.5 (I) 68.1 68.2 (X) 69.6 (X)	65.2 (I) 65.6 (I) 65.6 (I) 67.4 ± 0.4	65.5 ± 0.2 66.0 (X) 66.1 (X) 67.1 (X)

Quoted doses are normalised to the reading at d_max._ Cubic spline polynomials were used to generate extrapolated and interpolated doses, which are indicated by the symbols (X) and (I), respectively

### Comparison of experimental measurements to calculations

Geant4 beam entry dose values at a 0.05 mm WED, recorded (9.3 ± 0.4)%, (10.5 ± 0.4)%, (11.1 ± 0.4)%, 12.0 ± 0.4)%, and (13.1 ± 0.4)% for 1 × 1 cm^2^, 3 × 3 cm^2^, 5 × 5 cm^2^, 10 × 10 cm^2^, and 22 × 22 cm^2^ field sizes, respectively. Geant4 data interpolated to a depth suitable for skin-dose assessment at the point of beam entry was found to agree, within 1.0% (percent difference), to MO*Skin*™ measured dose at a 0.07 mm WED, for all field sizes. The greatest percent change between MO*Skin*™ and Geant4 interpolated doses at a 0.07 mm WED was found to be − 10.1% across all field sizes investigated.

Monaco TPS reported near-surface dose values at the beam entry, 0.50 mm WED, were (26.3 ± 0.2)%, (24.0 ± 0.2)%, (24.2 ± 0.2)%, (24.9 ± 0.2)%, and (26.1 ± 0.2)% for 1 × 1 cm^2^, 3 × 3 cm^2^, 5 × 5 cm^2^, 10 × 10 cm^2^, and 22 × 22 cm^2^ field sizes, respectively. The largest percent difference and change between the 0.07 mm WED MO*Skin*™ results and 0.50 mm WED TPS estimated doses was − 16.4% and − 166.4%, respectively, for all field sizes. The largest percent difference and change between 0.07 mm WED MO*Skin*™ measurements and TPS data extrapolated to an equivalent depth suitable for skin-dose assessment was − 2.8% and − 28.9%, respectively, across all fields. The maximum percent difference between film measurements and Monaco TPS extrapolated dose at a 0.34 mm WED was 8.4% for all field sizes investigated. Similarly, the maximum percent difference between film and Geant4 interpolated doses at a 0.34 mm WED was − 9.2%.

At the phantom exit, the largest percent difference and change of MO*Skin*™ measurements at a 199.67 mm WED to Geant4 estimated dose at a 199.95 mm WED, was -5.0% and − 8.0%, respectively, across all field sizes. Percent difference within 3.1% was found between 199.67 mm WED MO*Skin*™ measurements and 199.50 mm WED Monaco TPS estimated exit dose over all fields. For film data at a 166.66 mm WED, the largest percent differences to 199.50 mm WED TPS and 199.95 mm WED Geant4 estimates exit surface doses were 3.3% and 2.7%, respectively. Table 2 incorporates additional evaluations of exit dose at equivalent WEDs and demonstrates that the dose fluctuation across the final 0.5 mm of the phantom is comparatively stable in comparison to the dose variation within the initial 0.5 mm of the phantom where the beam is incident.

For fields larger than 1 × 1 cm^2^, Geant4 PDDs showed a field size-dependent dose increase at the phantom exit due to the ERE. Between 187.0 and 200.0 mm, dose increases of 11.2%, 14.2%, 16.4%, and 17.9% for the 3 × 3 cm^2^, 5 × 5 cm^2^, 10 × 10 cm^2^, and 22 × 22 cm^2^ field sizes, respectively, were found using Geant4 calculations. Similarly, increases of 11.7%, 14.7%, 16.0%, and 16.7% were found using Monaco TPS calculations. The largest increase in exit dose between 187.0 mm and 200.0 mm reported using MO*Skin*™ and film measurements was 15.4% for the 10 × 10 cm^2^ field and 15.7% for the 5 × 5 cm^2^ field, respectively. For all field sizes investigated, Monaco TPS and Geant4 PDD curves agreed, within 2.8% (percent change), for depths between 0.5 mm and 199.50 mm. Excluding the 1 × 1 cm^2^ field size, Monaco TPS and Geant4 PDD curves agreed within 1.7%. An improved agreement, within 0.9%, between the Monaco TPS and Geant4 PDD curves was found between 0.5 mm and 187.0 mm depths for the 3 × 3 cm^2^, 5 × 5 cm^2^, 10 × 10 cm^2^, and 22 × 22 cm^2^ field sizes.

### MO*Skin*™ comparison to pre-existing OSLD measurements

MO*Skin*™ surface and near-surface doses comparisons to pre-existing OSLD work performed by Kim et al. [17] for a 5 × 5 cm^2^, 10 × 10 cm^2^, and 22 × 22 cm^2^ field size, are shown in Tables 3 and 4. The largest percent difference and change at the beam entry between surface MO*Skin*™ measurements and pre-existing OSLD measurements was − 4.6% and − 38.9%, respectively, for all three field sizes. At a near equivalent WED of 0.16 mm, the largest percent difference and change of MO*Skin*™ measured beam entry doses to OSLD measurements were − 1.1% and − 6.4%, respectively, across the three field sizes.

**Table 3:** Comparison of entry dose measured with the MO*Skin*™, normalised to the reading at 13.0 mm, and published OSLD data*, normalised to the reading at 15.0 mm

Field size (cm^2^)	Entry (% D_max_)						
	(1)	(2)	(3)	(4)	(5)	(6)	(7)
	MO*Skin*™ WED = 0.07 mm	MO*Skin*™ WED = 0.17 mm	OSLD* WED = 0.16 mm	% Difference	% Change	% Difference	% Change
5 × 5	11.3 ± 0.6	15.3 ± 1.1	15.7	− 4.4	− 38.9	− 0.4	− 2.5
10 × 10	12.9 ± 1.0	16.2 ± 0.8	16.7	− 3.8	− 29.5	− 0.5	− 3.1
22 × 22	13.4 ± 1.0	16.9 ± 0.7	18.0	− 4.6	− 34.3	− 1.1	− 6.4

[17]. (1) MO*Skin*™ measured skin-equivalent dose; (2) MO*Skin*™ measured near-surface dose at a WED of 0.17 mm; (3) Published OSLD data; (4) percent (%) difference between columns (1) and (3); (5) % change between columns (1) and (3); (6) % difference between columns (2) and (3); (7) % change between columns (2) and (3)

**Table 4:** Exit MO*Skin*™ dose, normalised to the reading at 13.0 mm, compared with published OSLD data*, normalised to the reading at 15.0 mm [17]

Field size (cm^2^)	Exit (% D_max_)						
	(1)	(2)	(3)	(4)	(5)	(6)	(7)
	MO*Skin*™ WED = 199.67 mm	MO*Skin*™ WED = 199.84 mm	OSLD* WED = 200.16 mm	% Difference	% Change	% Difference	% Change
5 × 5	54.9 ± 2.0	59.8 (I)	56.0	− 1.1	− 2.1	3.8	6.4
10 × 10	63.9 ± 1.6	63.9 (I)	62.8	1.1	1.7	1.1	1.7
22 × 22	62.4 ± 3.0	63.3 (I)	63.4	− 1.0	− 1.6	− 0.1	− 0.2

(1) MO*Skin*™ measured dose at a 199.67 mm WED; (2) MO*Skin*™ dose interpolated to a WED of 199.84 mm; (3) Published OSLD data, (4) percent (%) difference between columns (1) and (3); (5) % change between columns (1) and (3); (6) % difference between columns (2) and (3); (7) % change between columns (2) and (3)

At the exit surface of the 200.0 mm thick phantom, MO*Skin*™ measurements when positioned at a 199.67 mm WED were within 1.1% (percent difference) to pre-existing OSLD measurements for 5 × 5 cm^2^, 10 × 10 cm^2^, and 22 × 22 cm^2^ field sizes. The largest percent change between the 199.67 mm WED MO*Skin*™ doses and OLSD measurement was − 2.1%, across all field sizes. The largest percent difference and change between MO*Skin*™ data extrapolated to a WED of 199.84 mm and OSLD measurements was 3.8% and 6.4%, respectively, for all three fields.

## Discussion

The impact of the magnetic field on the dose distribution poses a significant challenge, particularly for dose assessment at interfaces between materials of differing densities, such as air-tissue and tissue-air interfaces [2, 9]. A reduction of electron contamination at the entry surface due to the transverse magnetic field and the ERE at the exit surface creates heterogeneous dose regions where the accuracy of dosimetry is limited by the size of the SV.

The findings from this investigation demonstrate a decrease in the field size dependence on surface dose than is typically seen for a conventional linac. For a 6 MV FFF beam, it has been reported that the surface dose increases linearly with field size by approximately 0.9% per cm^2^ (for a square field) as measured using EBT-2 film (Gafchromic, International Specialty Products, Wayne, NJ) [48]. In contrast, this study found that for a 7 MV FFF 1.5 MR-linac, the surface dose increased by approximately 0.2% per cm^2^ as measured by the MO*Skin*™ detector and normalised relative to D_max_ for each corresponding field. A decrease in the dependency of field size on surface dose in a 1.5 T transverse MR-linac has been documented in previous studies [16, 17]. This can be attributed to two factors: the removal of the flattening filter, which serves as a major contributor of contaminant electrons, and the presence of a transverse magnetic field, which effectively removes contaminant electrons from the primary beam. The reduction in contaminant electrons enhances skin sparing, but also leads to an increase in the gradient of the build-up region. This raises the possibility of increased dosimetry errors due to volume averaging effects. In this study it was determined there was a lack of consistency between MO*Skin*™, film, Geant4, and Monaco TPS surface doses at the beam entry. This inconsistency can be attributed to the size of the sensitive or scoring volume and its relative position within the build-up region.

In the build-up region, there was good agreement between MO*Skin*™ measured and Geant4 estimated surface and near-surface doses, as shown in Figs. 3 and 4. Geant4 doses interpolated to the depth recommended for skin dose assessment agreed within 1.0% (percent difference) to MO*Skin*™ skin-equivalent surface doses (Table 1). There was less favourable agreement between film and TPS doses extrapolated to a depth suitable for skin dose assessment, with a maximum percent difference of 14.3% and 2.8%, respectively, to 0.07 mm WED MO*Skin*™ measurements for all field sizes investigated.

The 1.0 mm dose grid size of the TPS, being relatively large in comparison to the recommended depth for skin dose assessment [33], leads to an overestimation of the surface dose without extrapolation to the recommended depth. For practical purposes, the TPS-determined surface dose is often considered equivalent to the dose deposited in the skin during the planning phase and generally, it isn’t feasible to scale the dose to the skin-specific depth for each patient plan. Similarly, utilizing the raw near-surface measured film data, which was irradiated in a parallel orientation relative to the primary beam, for skin dose assessment would result in a significant overestimation of the dose. The magnitude of this overestimation was determined to be as high as 17.5% (percent difference) to the MO*Skin*™ skin-equivalent doses, across all fields investigated.

The MO*Skin*™ surface and near-surface doses were compared with the pre-existing OSLD measurements performed by Kim et al. [17] for equivalent radiation conditions. The comparison between the two dosimeters can be found in Tables 3 and 4. Kim et al. [17] also provided extrapolated surface doses for depths of 0.0 mm and 0.07 mm. However, it is deemed more appropriate to consider the OSLD data acquired experimentally at the shallowest depth of 0.16 mm due to the potential uncertainties associated with extrapolating from only two data points. Previous investigations that utilized OSLDs and TLDs for the determination of skin dose through extrapolation employed a minimum of three different TLD thicknesses [49, 50] and OSLD configurations [51]. These studies also address the limitations of utilizing a linear fit in the build-up region for extrapolated predictions of skin dose [50, 51]. At a near-equivalent WED, beam entry MO*Skin*™ measurements were in good agreement, within 1.1% (percent difference) to pre-existing OSLD measurements performed at 5 × 5 cm^2^, 10 × 10 cm^2^, and 22 × 22 cm^2^ field sizes. In comparison to the surface MO*Skin*™ measurements, the raw and uncorrected 0.16 mm WED OSLD values would not provide an accurate assessment of skin dose at the recommended depth of 0.07 mm.

In accordance with the findings of Kim et al. [17], marked differences were observed between the results of this study and the film surface doses reported by Woodings et al. [16]. The percent differences of 9.9% and 7.7% between the film results from this investigation and those of Woodings et al. [16], when comparing the results for a 5 × 5 cm^2^ and 10 × 10 cm^2^ field size, respectively, under equivalent irradiation conditions, suggest disparities in either the experimental set-up or analysis. A higher scan resolution of 254 DPI was also considered for the film analysis however it introduced large and incoherent variability along the PDD curves. Ordinarily, parallel film irradiation can produce smooth and continuous depth dose curves. However, in this investigation with the film placed in the transverse plane between four abutting solid water blocks that had varying surface flatness, air gaps were unavoidable despite water being used on the film and solid water block surfaces. Irradiating films parallel to the beam, in the coronal plane, within solid water blocks of dimensions 20 × 30 × n cm^3^ would minimise this issue; however, these were not available during this investigation. The continuous depth dose film data demonstrates the sensitivity of air gaps on film dosimetry in the MR-linac despite following a protocol that yields an uncertainty below 3.0% [47]. Perpendicular film irradiation has the potential to address the overestimation of surface dose obtained by irradiating the film in a parallel orientation. This approach eliminates the inaccuracies associated with parallel film irradiation, including overhanging film outside of the phantom and the air gaps within the phantom that are influenced by the ERE. It is important to note, however, that parallel film irradiation is limited in its ability to provide continuous depth dose data, and as such, was not included in the present study.

For beam exit dosimetry where the MO*Skin*™ was placed at the exit surface of the phantom and orientated face-up relative to the primary beam direction, the 0.4 mm thick silicon substrate included in the detector packaging [32], was exposed to air. Hence, there was the possibility that additional photon interactions within the thin silicon substrate, relative to water, could be produced and escape into the air, resulting in a larger measured exit dose, related to the ERE. The mass attenuation coefficient ratio of silicon to water is approximately equivalent above 0.2 MeV, hence the probability of photon interactions in the materials is approximately equal at these energies. Below this energy, the probability of photon interactions in silicon drastically increases and it cannot be considered water equivalent [45, 52]. The Elekta Unity system has an average photon beam energy of 2.11 MeV and a peak beam energy of 0.96 MeV [53, 54]. Of the total number of photon histories, few photons with energy below 0.2 MeV exist, hence no additional secondary electrons are expected, with the introduction of the silicon substrate, to contribute to the exit surface dose measured using the MO*Skin*™ detector. A maximum percentage difference of 5.8% between MO*Skin*™ measurements and film, Geant4, and TPS estimated beam exit doses provides evidence that the silicon substrate does not generate significant additional ERE. However, it can be speculated to a certain extent that the silicon substrate affects the recorded dose as it attenuates low energy returning electrons.

The results presented in Table 4 demonstrate that the 199.67 mm WED MO*Skin*™ measurements at the beam exit are in agreement with pre-existing OSLD measurements acquired by Kim et al. [17] for field sizes of 5 × 5 cm^2^, 10 × 10 cm^2^ and 22 × 22 cm^2^, within the bounds of experimental uncertainty. The improved concordance between the two detectors at the exit, despite slight variations in measurement locations, can be attributed to the shallower dose gradient compared to the beam entrance. The extrapolation of the MO*Skin*™ dose to be consistent with the rear build-up thickness traversed by returning electrons, as measured using OSLDs, did not improve agreement across the three field sizes. This was particularly pronounced for the 5 × 5 cm^2^ field MO*Skin*™ dose, which was lower than expected and thus inadequate to be used as a sample point for the extrapolation fit. To achieve a more accurate measurement of the ERE skin dose, it is advisable to use a face-up MO*Skin*™ relative to the exit surface. This orientation would help mitigate the potential attenuation of returning electrons through the substrate layer positioned at the rear of the device. With a face-up orientation of the MO*Skin*™ at the exit surface, returning electrons would traverse a WET of 0.07 mm to reach the SV as opposed to a 0.9 mm WET through the silicon substrate encountered in the face-down orientation [32] used throughout this investigation.

While the beam entry dose for the Elekta Unity is relatively unaffected by the field size, the exit dose and the net dose increase in the final 13.0 mm of the phantom at the beam exit were found to depend on the field size for field sizes larger than 1 × 1 cm^2^ (Figs. 3 and 4). This can be explained considering that for small fields, CPE conditions are not established [26] and a shift in the maximum exit dose away from the central axis occurs [11]. Between 187.0 and 200.0 mm depths within the phantom, Geant4 simulations reported the largest dose increase of 17.9% which corresponded to the 22 × 22 cm^2^ field size. The largest increase in exit dose due to ERE measured using the MO*Skin*™ was 15.4% for the 10 × 10 cm^2^ field size which can be attributed to underreported MO*Skin*™ doses at the exit surface that is particularly evident for the 3 × 3 cm^2^, 5 × 5 cm^2^, and 22 × 22 cm^2^ fields. Though the ERE has the potential to deposit additional dose at tissue-air boundaries, using matched-opposing fields or intensity-modulated radiation therapy (IMRT) can minimise such effects [55]. In addition, a water equivalent bolus of 1.0 cm placed at the exit surface can be used to significantly reduce ERE dose [11]; however, this method is not often used clinically as sufficient control can be achieved with TPS optimisation.

The MO*Skin*™ has previously been proven effective for in-vivo dosimetry (IVD) [28, 33, 56]; and presents the potential for real-time MR-linac dosimetry. However, the current readout cable design, while facilitating measurements and real-time readouts, also causes MRI image distortions that impede its use for real-time IVD on MR-linac systems. To address this limitation, the Centre of Medical Radiation Physics (CMRP) at the University of Wollongong, Australia is actively working to modify the existing cable design to avoid MR image noise and hence enable MO*Skin*™ real-time IVD during MR imaging. As of now, MO*Skin*™ dosimetry readout is only feasible when image sequences are not being acquired.

## Conclusion

The MO*Skin*™'s inherent design eliminates the application of correction factors and data extrapolation to a 0.07 mm WED, making it a convenient alternative to other dosimeters for ICRP-recommended skin dose assessment. In the present study, the Monte Carlo method, particularly the Geant4 toolkit, was employed as a benchmark for comparison with experimental measurements, due to its widespread recognition as a “gold standard” for accurate radiotherapy dose calculations. The results from this study verify the suitability of the MO*Skin*™ detector for transverse MR-linac dosimetry, particularly at beam entry and exit locations where the magnetic field can create steep dose gradients.

Geant4 dose calculations were able to validate the robustness of the Monaco TPS which agreed, within 2.8% (percent difference), to Geant4 results for all field sizes between 0.5 and 199.50 mm. Excluding the region influenced by the ERE, Geant4 results are consistent, within 0.9% (percent difference), to Monaco TPS depth doses between 0.5 and 187.0 mm for the 3 × 3 cm^2^, 5 × 5 cm^2^, 10 × 10 cm^2^, and 22 × 22 cm^2^ field sizes.

## Data Availability

All data relevant to this article can be made available upon request.
